# Supplementary material for: The effects of indoor temperature and humidity on local transmission of COVID-19 and how it relates to global trends
Source: PLoS One. 2022 Aug 10;17(8):e0271760. doi: 10.1371/journal.pone.0271760 (PMC9365153; doi:10.1371/journal.pone.0271760)
Supplement: S1 File — (DOCX) [file pone.0271760.s001.docx]

**(Full title) The effects of indoor temperature and humidity on local transmission of COVID-19 and how it relates to global trends**

**(Short title) Effects of indoor temperature and humidity on the spread of COVID-19**

**Supplementary Materials**

### **Supplementary Figure**

### **Evaporation experiment results**


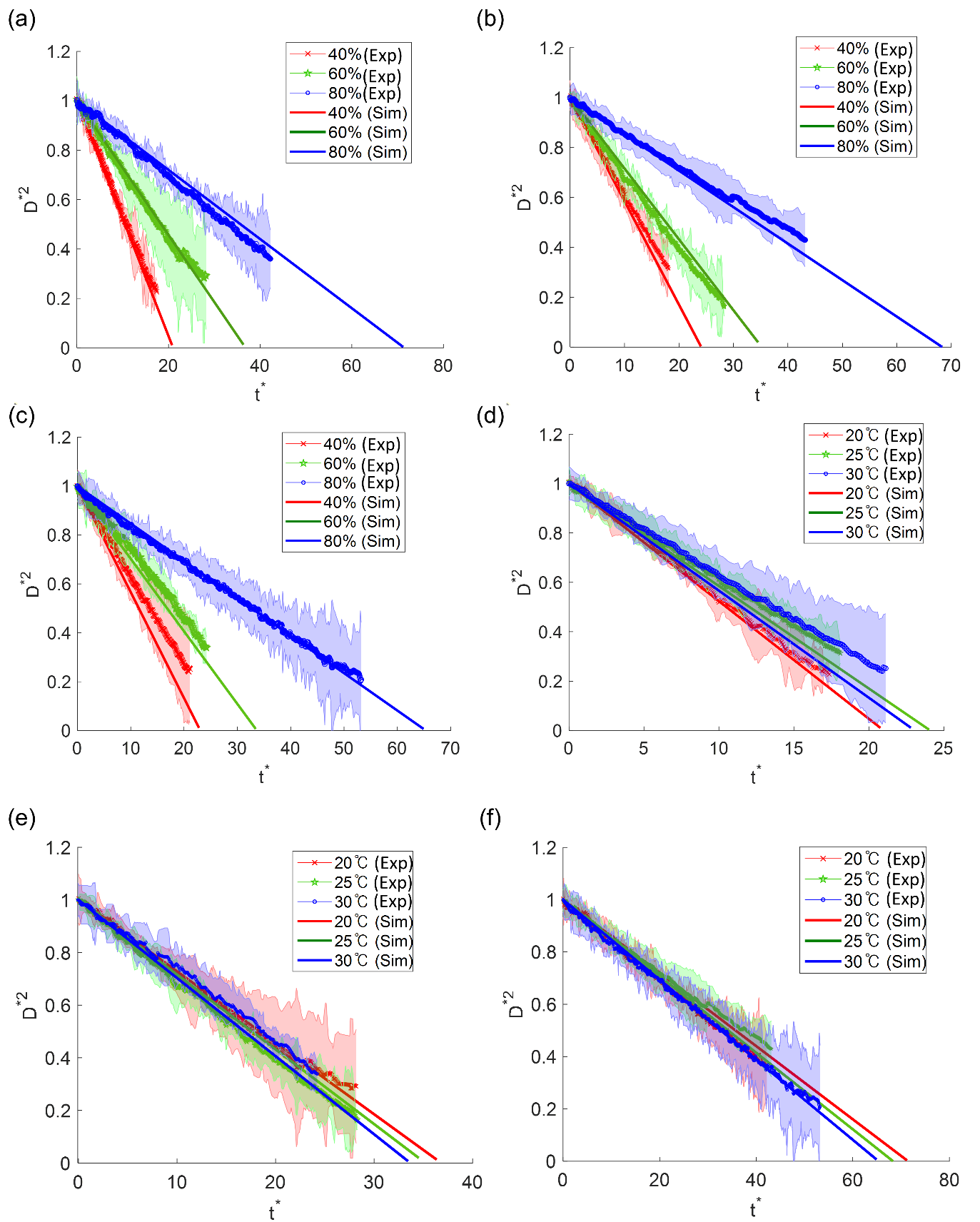


**Supplementary Figure 1. Droplet evaporation experiment results at various conditions with uncertainty at 95% confidence level. (a) T= 20℃ (b) T=25℃ (c) T= 30℃ (d) RH=40% (e) RH=60% (f) RH= 80%.**

### **Non-linear effect of relative humidity on COVID-19 transmission**


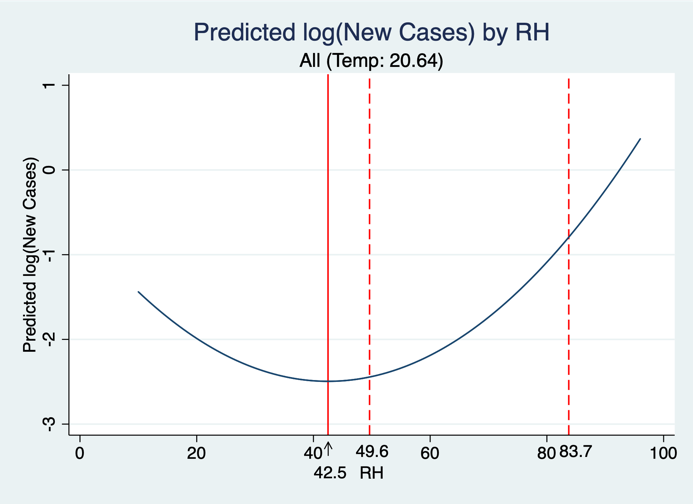


**Supplementary Figure 2. Estimated effect of monthly average RH on the natural logarithm of new and cumulative infection cases as of the last day of each month, fixing temperature (20.64℃). The solid line represents the lowest point, and the dashed line implies one standard deviation from the mean of RH.**

### **Supplementary notes**

### **Image processing methodology**

All of the videos were acquired at 100 fps, for up to 10 - 100 seconds. Thus, each video contains 1,000 - 10,000 images. Droplet diameter and location information can be obtained from these images. However, this information cannot be extracted for all images, because some microdroplets are out of focus due to out-of-plane motion. Therefore, the images were first screened based on their sharpness, using the Laplacian method^1^ to characterize the amount of blur. This process allows extraction of well-focused images. After the initial diameters and positions were obtained, image noise was removed. The droplet boundaries were then determined using the Canny edge detection method^2^. Finally, the diameter and central position could be accurately calculated.

### **Acoustic levitator streaming effect and associated Sherwood number change**

### When an acoustic levitator is used to investigate droplet evaporation, a vortex is created around the droplet due to the pressure wave from the microphone, and the droplet oscillates a bit. This acoustic streaming effect changes the overall Sherwood number^3-5^. In order to compare the experimental and theoretical Sherwood number, a correction must be made to the theoretical value. The corrected spatially averaged Sherwood number can be written as^6,7^

### $\left\langle\bar{Sh} \right\rangle=1.89\frac{A_{0e}}{\rho_{0}c_{0}\sqrt{\omega\mathcal{D}_{o}}}$ (1)

### $Sound Pressure Level (SPL)=20{log}_{10}\left( A_{0e} \right)+74$ (2)

### where *Sh* is the Sherwood number, *A_Oe_* is the incident acoustic field, *c_0_* is the sound velocity, *ρ_0_* is the unperturbed gas density, *ω* is the microphone frequency, and $\mathcal{D}_{o}$ is the diffusivity in air. We also need to know the pressure of the acoustic field to calculate *A_Oe_*, as given by equation (2). We measured the central frequency and sound pressure level (SPL) of the acoustic levitator using a 1/4^’’^ free-field microphone Type 4939 (4 Hz ~ 100 kHz). Since the SPL was not measurable at high voltage with the microphone, the SPL was first measured at low voltage using superposition of the pressure waves, and then it was linear extrapolated to 25 V for the actual experiment^6^. The frequency of the speakers was confirmed to be 40 kHz (as given by the manufacturer), and the SPL was estimated to be 156 dB at our experimental voltage level of 25 V.

### **Data and Econometric Analysis**

### **3.1. Definition of Country Groups for Cross-Sectional Analyses**

### As cross-sectional data includes each geographical location once, we cannot capture country-specific characteristics by exploiting country-fixed effects for countries that have only country-level information (i.e., 124 countries). For this reason, we introduce the following two variables to classify countries and control for region-specific effects using the limited data available.

### First, we create a dummy variable that specifies whether the given location belongs to the southern hemisphere, based on geography information from the COVID-19 Open Data. The variable takes the value of one if the latitude of the given location has a negative value, and zero otherwise. In addition, following the World Bank’s country classification, we construct a variable which classifies the countries into seven different geographical regions – East Asia and Pacific, Europe and Central Asia, Latin America and Caribbean, Middle East and North Africa, North America, South Asia, Sub-Saharan Africa. Note that five countries in our sample (i.e., Brazil, Colombia, the Democratic Republic of the Congo, Indonesia, and Kenya) have multiple locations in our data, and some of them are classified to be within the southern hemisphere, and the rest are within the northern hemisphere (depending on their latitude).

### **3.2. Summary Statistics**

### Supplementary Table 1 shows summary statistics of our global COVID-19 data, restricted to our baseline model in column (1) panel A of Table 1 in the manuscript. Since the number of locations in a given country varies, statistics are weighted by the reciprocal of the number of observations for a given country and month, which allows all countries to be treated equally.

### **3.3. Econometric Analysis of Global COVID-19 data**

### Supplementary Tables 2 and 3 present detailed results of Panel A and B in Table 1 of the manuscript, respectively. As shown in column (1) of Supplementary Table 2, implementing a single unit more stringent policy is correlated to a 0.060 increase in $log (Y_{l,c, t})$ for a given location and month. This implies that adopting a single unit more stringent policy corresponds to a 6.0% average increment in number of new COVID-19 patients. Similarly, single unit greater health and economic support are associated with 2.5% and 0.8% reduction, respectively. All coefficients are statistically significant at the 1% level. Some readers may be puzzled by the positive coefficient of policy stringency. A positive coefficient may be possible, because a government might have introduced a more stringent policy in response to its expectation that COVID-19 would be rapidly spreading. Thus, the positive coefficient captures a mixture of the causal effect of the stringent policy on the spread as well as the trend of rapid increase in new COVID-19 cases. The remaining results reflect correlations of precipitation and air pollutant concentration levels. These results are robust even after including the cumulative number of previous cases as an additional explanatory variable, as shown in column (2), or exploiting cumulative confirmed cases as the dependent variable (Supplementary Table 3).

To ensure the robustness of our finding, we employed various alternative assumptions in our regression model and compared the results with our baseline finding. For example, some studies found that pollutants may have impact on health outcomes if they exceed a certain threshold^8,9^. To incorporate this possibility, we counted the number of days in which daily PM_10_ level (or PM_2.5_) exceeded the WHO criteria (i.e., PM_10_: 45 μg/m^3^, PM_2.5_: 15 μg/m^3^) and used the fraction of such days in a month as an explanatory variable, instead of using the monthly average PM_10_ level. However, the coefficients of temperature and RH, which are of key interest in this paper, quantitatively remain stable. Specifically, the coefficient of temperature in our baseline model (Column (1) of Supplementary Table 2) was -0.027 (p-value < 0.001) while that in the alternative model was -0.024 (p-value < 0.001). The coefficient of RH was -0.009 (p-value < 0.001) in our baseline model and -0.010 in the alternative model (p-value < 0.001). Also, some studies report the importance of DTR (difference between the daily maximum and minimum temperature) on the spread of viruses.^10-12^ To examine the robustness of our main finding, we included the monthly average DTR for each location in our model. Our key coefficients remain quantitatively the same, or even strengthened, as the coefficient of temperature was -0.038 (p-value < 0.001) and that of RH became -0.017 (p-value < 0.001).

### Our baseline models assume that the role of temperature and relative humidity on COVID-19 transmission remains the same across countries and months, for the sake of simplicity and also to be in line with previous studies.^13-16^ To check the possibility of different effects depending on location and period, we divide our sample by quarters (January to March, April to June, July to September, and October to December) and region across the world (followed by the World Bank’s classification to categorize countries into seven geographical areas). Supplementary Table 4 reports these results. In column (1), we report our baseline case in which we regress the logarithm of new cases on the logarithm of past cases as well as country fixed effects and other controls (i.e., Column (2) of Table 1 in the manuscript). Columns (2) to (5) report the estimated coefficients for each quarter. Although the estimated magnitudes vary, the sign of the estimates and their statistical significance remains the same across all quarters.

### We separately estimate our regression model by each region, similar to the quarterly regression. Supplementary Table 5 shows these results. As shown in columns (2) to (5), four regions – East Asia & the Pacific, North America, Latin America & the Caribbean, and Sub-Saharan Africa – show a statistically significant negative coefficient for temperature as well as relative humidity, which matches our baseline results in column (1). However, for two regions – Europe & Central Asia, and South Asia – the sign of one coefficient is negative while the other is positive, while the signs of both coefficient estimates are positive for the Middle East & North Africa.

### Although we cannot rule out other possibilities, we suggest two possible causes that may account for these differences. One is the regional variation in temperature and relative humidity. As shown in the bottom two rows of Supplementary Table 5, the average temperature in Europe & Central Asia is only 11.72, quite a bit lower than the sample average of 20.64, while the average RH in the Middle East & North Africa is only 44.45, quite a bit lower than the sample average of 66.68.

### If temperature and relative humidity affect the transmission of COVID-19 in a non-linear fashion, then this may also account for the differences across regions. To test this possibility, we extend our baseline model by including squared values of temperature and relative humidity. By doing so, we allow for the possibility that the effect could be positive or negative, depending on the value at which the overall effect of temperature or relative humidity is evaluated. Supplementary Table 6 shows the results. In column (1), we report the results from all regions, while columns (2) to (4) report the results for the three regions that previously showed different patterns. Column (1) shows that the coefficients of temperature and temperature-squared are negative and positive, respectively, suggesting a U-shaped relationship between temperature and the number of new COVID-19 cases. A similar pattern is found for RH as well. As shown in columns (2) to (4), the signs of temperature and temperature-squared are all the same as those in column (1), consistent with our baseline results. For the coefficients of RH and RH-squared, two regions - Europe & Central Asia and South Asia – show the same signs as those in column (1). However, the Middle East & North Africa region shows opposite signs for RH and RH-squared. We suspect that the much lower value of RH in this region compared to all other regions may account for this finding.

### **3.4. Econometric Analysis of CFD simulation results**

### Supplementary Table 7 reports additional results of Panel A in Table 2 of our manuscript, including coefficients of dummy variables for each initial droplet size. Since we set the initial droplet size of 90 μm as a reference and therefore omitted this group, the coefficient for the dummy variable should be interpreted as a relative term. For instance, column (1) shows that droplets which have an initial size of 110 μm drop to the ground on average 3.2 sec sooner than those which have an initial size of 90 μm, under identical temperature and RH settings. Likewise, initially large droplets tend to fall to the ground sooner and travel less, if they drop to the ground before fully evaporating, as illustrated in columns (1) – (2). In contrast, if we examine the droplets that fully evaporate before they hit the ground, shown in columns (3) to (5), we find that the droplet size and outcome variables are sometimes monotonically related, but not always. For example, in column (3), the elapsed time is shorter for initially smaller droplets. In other words, the elapsed time is positively correlated with the initial droplet size. Likewise, the final height is negatively correlated with the initial droplet size, as evidenced in column (5). However, for the final distance shown in column (4), there is no clear correlation between initial drop size and final distance travelled.

### **3.5. Conversion process of outdoor data to indoor data**

### The process of converting outdoor data to indoor data is explained below.

First, we identified the regions with the highest number of confirmed COVID-19 cases. We found that most countries with many confirmed cases were divided into two climates: tropical and temperate climate.

Next, we used the tropical and temperate zone data conversion correlation based on previous studies (Nguyen et al. 2014 and Nguyen and Dockery 2016). In the case of tropical zone, the Sao-Paulo correlation was used. In the case of temperate zone, Atlanta, Athens, Boston, and Dublin correlation was used. Since the correlation tendencies of temperate zones were similar, the correlations of these regions were averaged. The average correlation coefficient turned out to be similar with the Boston local correlation.

### Finally, we convert outdoor temperature and relative humidity into indoor conditions based on the average correlation coefficient. Temperature and absolute humidity was converted directly between indoors and outdoors, but there was no direct correlation for relative humidity. Therefore, we converted the absolute humidity from outdoors to indoors, and then calculated the indoor relative humidity using the converted indoor temperature.

Supplementary Table 1. Summary Statistics

|  | Num. obs. | Mean | Std. Dev. | Min | Max |
| --- | --- | --- | --- | --- | --- |
| Cum. cases | 9,414 | 33,597 | 109,935 | 1 | 3,421,720 |
| New cases | 9,414 | 8,875 | 31,600 | 1 | 1,067,379 |
| Outdoor Temperature (℃) | 9,414 | 20.52 | 9.566 | -44.94 | 39.70 |
| Outdoor RH (%) | 9,414 | 66.57 | 16.98 | 10.08 | 95.58 |
| Indoor Temperature (℃) | 8,391 | 22.62 | 2.757 | 18.90 | 30.48 |
| Indoor RH (%) | 8,391 | 56.58 | 12.76 | 21.66 | 85.69 |
| Precipitation (cm) | 9,414 | 0.017 | 0.092 | 0.000 | 2.366 |
| CO (g/m^2^) | 9,414 | 0.937 | 0.282 | 0.352 | 2.498 |
| SO_2_ (g/m^2^) | 9,414 | 0.005 | 0.010 | 0.000 | 0.149 |
| NO (pg/m^2^) | 9,414 | 1.337 | 1.729 | 0.054 | 68.87 |
| NO_2_ (g/m^2^) | 9,414 | 0.005 | 0.005 | 0.001 | 0.097 |
| PM_2.5_ (μg/m^3^) | 9,414 | 27.38 | 38.06 | 0.192 | 266.3 |
| PM_10_ (μg/m^3^) | 9,414 | 82.99 | 125.3 | 0.327 | 844.6 |
| Stringency | 9,414 | 57.76 | 20.63 | 0.000 | 100.0 |
| Health | 9,414 | 54.17 | 16.23 | 0.000 | 91.35 |
| Economic Support | 9,414 | 46.59 | 30.13 | 0.000 | 100.0 |

***Note:*** We exclude observations where monthly new confirmed cases are zero. All results are weighted by the reciprocal number of observations of each country for each month. Outdoor temperature and relative humidity are converted to indoor conditions using correlations from Nguyen et al. (2014).

Supplementary Table 2. Effects of temperature and relative humidity on the logarithm of monthly new COVID-19 cases across 174 countries from Jan. 2020 to Feb. 2021

| Period | 2020/1 to 2021/2 | 2020/2 to 2021/2 | 2020/3 | 2021/2 | 2020/3 | 2021/2 |
| --- | --- | --- | --- | --- | --- | --- |
| Model | Baseline | Baseline +  Past cases | OLS | OLS | Weighted OLS + Group FE | Weighted OLS + Group FE |
|  | (1) | (2) | (3) | (4) | (5) | (6) |
|  |  |  |  |  |  |  |
| Temperature | -0.027*** | -0.038*** | -0.021 | -0.008 | 0.044*** | -0.083*** |
|  | (0.000) | (0.000) | (0.014) | (0.013) | (0.000) | (0.001) |
| RH | -0.009*** | -0.011*** | 0.007 | 0.012 | -0.017*** | -0.016*** |
|  | (0.000) | (0.000) | (0.010) | (0.010) | (0.000) | (0.000) |
| Stringency | 0.060*** | 0.049*** | -0.009 | 0.127*** | -0.013*** | 0.022*** |
|  | (0.000) | (0.000) | (0.030) | (0.032) | (0.000) | (0.001) |
| Health Support | -0.025*** | -0.031*** | 0.027 | -0.167*** | 0.046*** | 0.006*** |
|  | (0.000) | (0.000) | (0.036) | (0.038) | (0.000) | (0.001) |
| Econ. Support | -0.008*** | -0.009*** | 0.029*** | 0.028*** | 0.015*** | -0.013*** |
|  | (0.000) | (0.000) | (0.008) | (0.009) | (0.000) | (0.000) |
| Precipitation | -0.080*** | -0.333*** | 1.220** | 0.648 | 0.381*** | -0.866*** |
|  | (0.004) | (0.004) | (0.618) | (0.637) | (0.008) | (0.025) |
| CO | 0.468*** | 0.406*** | -2.085*** | -2.020*** | 0.277*** | 0.360*** |
|  | (0.003) | (0.003) | (0.622) | (0.690) | (0.008) | (0.014) |
| SO_2_ | 23.64*** | 12.91*** | -123.1*** | -77.24** | 3.445*** | 5.699*** |
|  | (0.070) | (0.062) | (27.71) | (34.09) | (0.503) | (0.964) |
| NO | -0.114*** | -0.089*** | -0.165 | -0.247 | -0.176*** | -0.619*** |
|  | (0.001) | (0.000) | (0.126) | (0.163) | (0.002) | (0.008) |
| NO_2_ | 82.82*** | 48.90*** | 236.1*** | 259.6*** | 105.5*** | 244.1*** |
|  | (0.283) | (0.247) | (67.62) | (77.46) | (1.330) | (3.061) |
| PM_2.5_ | -0.007*** | -0.008*** | 0.026 | 0.012 | -0.042*** | 0.005*** |
|  | (0.000) | (0.000) | (0.028) | (0.018) | (0.000) | (0.001) |
| PM_10_ | 0.001*** | 0.002*** | -0.001 | -0.003 | 0.000*** | -0.001*** |
|  | (0.000) | (0.000) | (0.003) | (0.002) | (0.000) | (0.000) |
| log(cum. case)(t-1) |  | 0.566*** |  |  |  |  |
|  |  | (0.000) |  |  |  |  |
| R-sq | 0.827 | 0.867 | 0.177 | 0.162 | 0.450 | 0.356 |
| Mean new cases | 8,875 | 9,520 | 971.3 | 6,859 | 971.3 | 6,859 |
| Obs. | 9,414 | 9,023 | 470 | 276 | 470 | 276 |

***Notes:*** The dependent variable is the natural logarithm of new infection cases as of the last day of each month. Columns (1) – (2) exploit panel data from January or February 2020 to February 2021, while columns (3) – (6) use cross-sectional data as of March 2020 and February 2021. Columns (1) – (2) include country fixed effects, month x calendar year x hemisphere fixed effects, and country-specific linear time trends, while columns (3) – (4) do not include any of these effects. Columns (5) – (6) include fixed effects of 8 country groups, which consist of geographically neighboring countries. Columns (1) – (2) and (5) – (6) are weighted by the reciprocal number of monthly observations for each country. Standard errors are in parentheses. The p-values are denoted as * for p < 0.10, ** for p < 0.05, and *** for p < 0.01.

Supplementary Table 3. Effects of temperature and relative humidity on the logarithm of monthly cumulative COVID-19 cases across 174 countries from Jan. 2020 to Feb. 2021

| Period | 2020/1 to 2021/2 | 2020/2 to 2021/2 | 2020/3 | 2021/2 | 2020/3 | 2021/2 |
| --- | --- | --- | --- | --- | --- | --- |
| Model | Baseline | Baseline +  Past cases | OLS | OLS | Weighted OLS + Group FE | Weighted OLS + Group FE |
|  | (1) | (2) | (3) | (4) | (5) | (6) |
|  |  |  |  |  |  |  |
| Temperature | -0.008*** | -0.012*** | -0.045*** | -0.013 | 0.035*** | -0.084*** |
|  | (0.000) | (0.000) | (0.010) | (0.013) | (0.000) | (0.001) |
| RH | -0.002*** | -0.001*** | -0.009 | 0.005 | -0.016*** | -0.022*** |
|  | (0.000) | (0.000) | (0.007) | (0.010) | (0.000) | (0.000) |
| Stringency | 0.029*** | 0.022*** | -0.105*** | 0.103*** | -0.063*** | 0.020*** |
|  | (0.000) | (0.000) | (0.020) | (0.031) | (0.000) | (0.001) |
| Health Support | -0.011*** | -0.018*** | 0.126*** | -0.142*** | 0.090*** | -0.006*** |
|  | (0.000) | (0.000) | (0.024) | (0.038) | (0.000) | (0.001) |
| Econ. Support | -0.002*** | -0.004*** | 0.006 | 0.030*** | -0.005*** | -0.003*** |
|  | (0.000) | (0.000) | (0.004) | (0.009) | (0.000) | (0.000) |
| Precipitation | 0.209*** | 0.097*** | 1.392*** | 0.444 | 0.371*** | -1.143*** |
|  | (0.003) | (0.002) | (0.449) | (0.632) | (0.008) | (0.025) |
| CO | 0.098*** | -0.003* | -0.715* | -1.802*** | -0.042*** | -0.010 |
|  | (0.002) | (0.001) | (0.380) | (0.665) | (0.007) | (0.014) |
| SO_2_ | 17.64*** | -2.701*** | -56.29*** | -71.29** | 29.89*** | -2.311** |
|  | (0.052) | (0.030) | (19.40) | (32.57) | (0.445) | (0.932) |
| NO | -0.136*** | -0.085*** | -0.471*** | -0.245 | -0.290*** | -0.482*** |
|  | (0.000) | (0.000) | (0.101) | (0.162) | (0.002) | (0.008) |
| NO_2_ | 87.62*** | 58.52*** | 371.0*** | 245.5*** | 180.6*** | 221.0*** |
|  | (0.200) | (0.115) | (50.76) | (76.27) | (1.197) | (2.982) |
| PM_2.5_ | 0.002*** | -0.004*** | -0.046*** | 0.029* | -0.042*** | 0.024*** |
|  | (0.000) | (0.000) | (0.014) | (0.017) | (0.000) | (0.000) |
| PM_10_ | -0.001*** | 0.001*** | 0.006*** | -0.004** | 0.001*** | -0.003*** |
|  | (0.000) | (0.000) | (0.002) | (0.002) | (0.000) | (0.000) |
| log(cum. case)(t-1) |  | 0.718*** |  |  |  |  |
|  |  | (0.000) |  |  |  |  |
| R-sq | 0.903 | 0.964 | 0.242 | 0.135 | 0.432 | 0.352 |
| Mean cum. cases | 31,641 | 34,987 | 935.3 | 100,589 | 935.3 | 100,589 |
| Obs. | 10,437 | 9,456 | 812 | 283 | 812 | 283 |

***Notes:*** The dependent variable is the natural logarithm of cumulative infection cases as of the last day of each month. Columns (1) – (2) exploit panel data from January or February 2020 to February 2021, while columns (3) – (6) use cross-sectional data as of March 2020 and February 2021. Columns (1) – (2) include country fixed effects, month x calendar year x hemisphere fixed effects, and country-specific linear time trends, while columns (3) – (4) do not include any of these effects. Columns (5) – (6) include fixed effects of 8 country groups, which consist of geographically neighboring countries. Columns (1) – (2) and (5) – (6) are weighted by the reciprocal number of monthly observations for each country. Standard errors are in parentheses. The p-values are denoted as * for p < 0.10, ** for p < 0.05, and *** for p < 0.01.

Supplementary Table 4. Subgroup Analysis by Quarter: Effects of temperature and relative humidity on the logarithm of monthly new COVID-19 cases and cumulative cases across 174 countries from Jan. 2020 to Feb. 2021

| Period | All | Q1 | Q2 | Q3 | Q4 |
| --- | --- | --- | --- | --- | --- |
|  | (1) | (2) | (3) | (4) | (5) |
| Temperature | -0.038*** | -0.026*** | -0.009*** | -0.029*** | -0.021*** |
|  | (0.000) | (0.001) | (0.000) | (0.000) | (0.000) |
|  | [0.000] | [0.000] | [0.000] | [0.000] | [0.000] |
| RH | -0.011*** | -0.005*** | -0.002*** | -0.008*** | -0.005*** |
|  | (0.000) | (0.000) | (0.000) | (0.000) | (0.000) |
|  | [0.000] | [0.000] | [0.000] | [0.000] | [0.000] |
| R-sq | 0.867 | 0.956 | 0.934 | 0.953 | 0.971 |
| Sample Mean |  |  |  |  |  |
| - Temperature | 20.64 | 16.24 | 21.80 | 23.70 | 17.93 |
| - RH | 66.68 | 66.97 | 63.25 | 66.42 | 70.26 |

***Notes:*** The dependent variable of the natural logarithm of new cases as of the last day of each month. We include country fixed effects, month x calendar year x hemisphere fixed effects, and country-specific linear time trends, and the logarithm of the cumulative cases. We apply weights by the reciprocal number of monthly observations for each country. Standard errors and p-values are in parentheses and square brackets, respectively. The p-values are denoted as * for p < 0.10, ** for p < 0.05, and *** for p < 0.01.

Supplementary Table 5. Subgroup Analysis by Region: Effects of temperature and relative humidity on the logarithm of monthly new COVID-19 cases and cumulative cases across 174 countries from Jan. 2020 to Feb. 2021

| Regional Group | All | East Asia & Pacific | North America | Latin America & Caribbean | Sub-Saharan Africa | Europe & Central Asia | South Asia | Middle East & North Africa |
| --- | --- | --- | --- | --- | --- | --- | --- | --- |
|  | (1) | (2) | (3) | (4) | (5) | (6) | (7) | (8) |
| Temperature | -0.038*** | -0.026*** | -0.024*** | -0.042*** | -0.087*** | 0.001*** | -0.012*** | 0.158*** |
|  | (0.000) | (0.000) | (0.001) | (0.000) | (0.000) | (0.000) | (0.001) | (0.000) |
|  | [0.000] | [0.000] | [0.000] | [0.000] | [0.000] | [0.000] | [0.000] | [0.000] |
| RH | -0.011*** | -0.001*** | -0.020*** | -0.007*** | -0.013*** | -0.002*** | 0.028*** | 0.013*** |
|  | (0.000) | (0.000) | (0.000) | (0.000) | (0.000) | (0.000) | (0.000) | (0.000) |
|  | [0.000] | [0.000] | [0.000] | [0.000] | [0.000] | [0.000] | [0.000] | [0.000] |
| R-sq | 0.867 | 0.813 | 0.943 | 0.886 | 0.845 | 0.889 | 0.879 | 0.889 |
| Sample Mean |  |  |  |  |  |  |  |  |
| - Temperature | 20.64 | 23.30 | 14.03 | 24.90 | 24.54 | 11.72 | 23.26 | 25.79 |
| - RH | 66.68 | 72.62 | 71.53 | 72.05 | 63.30 | 71.10 | 70.48 | 44.45 |

***Notes:*** The dependent variable of the natural logarithm of new cases as of the last day of each month. We include country fixed effects, month x calendar year x hemisphere fixed effects, and country-specific linear time trends, and the logarithm of the cumulative cases. We apply weights by the reciprocal number of monthly observations for each country. Standard errors and p-values are in parentheses and square brackets, respectively. The p-values are denoted as * for p < 0.10, ** for p < 0.05, and *** for p < 0.01.

Supplementary Table 6. Subgroup Analysis by Region with Non-Linear Effect of Temperature and RH: Effects of temperature and relative humidity on the logarithm of monthly new COVID-19 cases and cumulative cases across 174 countries from Jan. 2020 to Feb. 2021

| Regional Group | All | East Asia & Pacific | North America | Latin America & Caribbean |
| --- | --- | --- | --- | --- |
|  | (1) | (2) | (3) | (4) |
| Temperature | -0.054*** | -0.009*** | -0.146*** | -0.019*** |
|  | (0.000) | (0.000) | (0.002) | (0.001) |
|  | [0.000] | [0.000] | [0.000] | [0.000] |
| Temperature-squared | 0.001*** | 0.0004*** | 0.003*** | 0.004*** |
|  | (0.000) | (0.000) | (0.000) | (0.000) |
|  | [0.000] | [0.000] | [0.000] | [0.000] |
| RH | -0.085*** | -0.109*** | -0.038*** | 0.039*** |
|  | (0.000) | (0.000) | (0.001) | (0.000) |
|  | [0.000] | [0.000] | [0.000] | [0.000] |
| RH-squared | 0.001*** | 0.001*** | 0.001*** | -0.0003*** |
|  | (0.000) | (0.000) | (0.000) | (0.000) |
|  | [0.000] | [0.000] | [0.000] | [0.000] |
| R-sq | 0.870 | 0.891 | 0.882 | 0.891 |

***Notes:*** The dependent variable of the natural logarithm of new cases as of the last day of each month. We include country fixed effects, month x calendar year x hemisphere fixed effects, and country-specific linear time trends, and the logarithm of the cumulative cases. We apply weights by the reciprocal number of monthly observations for each country. Standard errors and p-values are in parentheses and square brackets, respectively. The p-values are denoted as * for p < 0.10, ** for p < 0.05, and *** for p < 0.01.

Supplementary Table 7. Effects of temperature and relative humidity on droplet transport

| Sample | **Dropped to the ground** | | **Fully evaporated** | | |
| --- | --- | --- | --- | --- | --- |
| Variables | Time Elapsed | Final Distance | Time Elapsed | Final Distance | Final Height |
|  | (1) | (2) | (3) | (4) | (5) |
|  |  |  |  |  |  |
| Temperature | 0.024 | 0.004 | -0.080 | -0.019* | -0.001 |
|  | (0.028) | (0.005) | (0.055) | (0.011) | (0.011) |
|  | [0.403] | [0.481] | [0.165] | [0.085] | [0.956] |
| RH | -0.033*** | -0.006*** | 0.194*** | 0.037*** | -0.011*** |
|  | (0.008) | (0.001) | (0.016) | (0.003) | (0.003) |
|  | [0.001] | [0.001] | [0.000] | [0.000] | [0.003] |
| 1(d0=50) |  |  | -4.087*** | 0.715*** | 0.587*** |
|  |  |  | (0.651) | (0.126) | (0.132) |
|  |  |  | [0.000] | [0.000] | [0.000] |
| 1(d0=70) |  |  | -1.605** | -0.461*** | 0.328** |
|  |  |  | (0.651) | (0.126) | (0.132) |
|  |  |  | [0.022] | [0.001] | [0.021] |
| 1(d0=110) | -3.212*** | -0.475*** | 2.479*** | 0.586*** | -0.334** |
|  | (0.429) | (0.078) | (0.781) | (0.151) | (0.158) |
|  | [0.000] | [0.000] | [0.004] | [0.001] | [0.046] |
| 1(d0=130) | -5.967*** | -0.910*** |  |  |  |
|  | (0.419) | (0.076) |  |  |  |
|  | [0.000] | [0.000] |  |  |  |
| 1(d0=150) | -7.762*** | -0.720*** |  |  |  |
|  | (0.419) | (0.076) |  |  |  |
|  | [0.000] | [0.000] |  |  |  |
| R-sq | 0.955 | 0.886 | 0.892 | 0.928 | 0.661 |
| Mean. Dep. | 4.915 (sec) | 1.132 (m) | 6.395 (sec) | 1.502 (m) | 1.020 (m) |
| Obs. | 26 | 26 | 28 | 28 | 28 |

***Notes:*** Three droplet transport characteristics (i.e. time elapsed, final distance and final height) from the CFD simulations are used as the dependent variable. All simulation results assume uniform ventilation flow. We use simulation data of droplets which hit the ground before they fully evaporate for columns (1) – (2), and droplets that fully evaporate for columns (3) – (5). Standard errors and p-values are in parentheses and square brackets, respectively. The p-values are denoted as * for p < 0.10, ** for p < 0.05, and *** for p < 0.01.

**References**

1. Bansal R, Raj G. Choudhury T. Blur image detection using Laplacian operator and Open-CV. International Conference System Modeling & Advancement in Research Trends (SMART) IEEE. 2016; 63-67.
2. Mousa A. Canny edge-detection based vehicle plate recognition. Int J Signal Process. 2012; 5: 1-8.
3. Kobayashi K, Goda A, Hasegawa K, Abe Y. Flow structure and evaporation behaviour of an acoustically levitated droplet. Phys Fluids. 2018; 30: 082105.
4. Pandey K, Prabhakaran D. Basu S. Review of transport processes and particle self-assembly in acoustically levitated nanofluid droplets. Phys Fluids. 2019; 31: 112102.
5. Sasaki Y, Kobayashi K, Hasegawa K, Kaneko A, Abe Y. Transition of flow field of acoustically levitated droplets with evaporation. Phys Fluids. 2019; 31: 102109.
6. Yarin A L, Pfaffenlehner M, Tropea C. On the acoustic levitation of droplets. J Fluid Mech. 1998; 356: 65-91.
7. Yarin A L, Brenn G, Kastner O, Rensink D, Tropea C. Evaporation of acoustically levitated droplets. J Fluid Mech. 1999; 399: 151-201.
8. Bontempi E. First data analysis about possible COVID-19 virus airborne diffusion due to air particulate matter (PM): The case of Lombardy (Italy). Environ Res. 2020; 186: 109639.
9. Rovetta A, Castaldo L. Relationships between Demographic, Geographic, and Environmental Statistics and the Spread of Novel Coronavirus Disease (COVID-19) in Italy. Cureus 2020; 12: e11397.
10. Islam A, Hasanuzzaman M., Azad M, Salam R, Toshi F, Khan M, Islam S, Alam G, Ibrahim S. Effect of meteorological factors on COVID-19 cases in Bangladesh. Environ Dev Sustain. 2021; 23: 9139–9162.
11. Yap T, Decker C, Preston D. Effect of daily temperature fluctuations on virus lifetime. Science of The Total Environment. 2021; 789: 148004.
12. Liu J, Zhou J, Yao J, Zhang X, Li L, Xu X, He X, Wang B, Fu S, Niu T, Yan J. Impact of meteorological factors on the COVID-19 transmission: A multi-city study in China. Science of The Total Environment. 2020; 726: 138513.
13. Sajadi M M, Habibzadeh P, Vintzaileos A, Shokouhi S, Miralles-Wilhelm F, Amoroso A. Temperature, humidity, and latitude analysis to estimate potential spread and seasonality of coronavirus disease 2019 (COVID-19). JAMA network open. 2020; 3: e2011834-e2011834.
14. Gupta S, Raghuwanshi G S, Chanda A. Effect of weather on COVID-19 spread in the US: A prediction model for India in 2020. Sci Total Environ. 2020; 728: 138860.
15. Menebo M M. Temperature and precipitation associate with COVID-19 new daily cases: A correlation study between weather and COVID-19 pandemic in Oslo, Norway. Sci Total Environ. 2020; 737: 139659.
16. Sharma P, Singh A K, Agrawal B, Sharma A. Correlation between weather and COVID-19 pandemic in India: An empirical investigation. J Public Aff. 2020; 20: e2222.
17. Nguyen J L, Schwartz J, Dockery D W. The relationship between indoor and outdoor temperature, apparent temperature, relative humidity, and absolute humidity. Indoor Air. 2014; 24: 103-112.
18. Nguyen J. L, Dockery D. W. Daily indoor-to-outdoor temperature and humidity relationships: a sample across seasons and diverse climatic regions. Int. J. Biometeorol. 2016; 60: 221-229.
