# Supplementary material for: The effects of indoor temperature and humidity on local transmission of COVID-19 and how it relates to global trends
Source: PLoS One. 2022 Aug 10;17(8):e0271760. doi: 10.1371/journal.pone.0271760 (PMC9365153; doi:10.1371/journal.pone.0271760)
Supplement: Attachment — Submitted filename: Response to reviewer #3.docx [file pone.0271760.s004.docx]

**Response to reviewer #3**

We thank the reviewer for the positive feedback. Comments are addressed below.

1. **Line 25: SARS-Cov-2 should be replaced as SARS-CoV-2.**

-Thank you for pointing this out. We revised the sentence as follows

Page 2 (Line 25)

During the COVID-19 pandemic, analyses on global data have not reached unanimous consensus on whether warmer and humid weather curbs the spread of the SARS-CoV-2 virus.

2. **Line 37: Again, please maintain the symmetry while writing SARS-CoV-2.**

- We went through the entire manuscript and fixed the notation.

Page 2 (Line 36)

Ever since the World Health Organization (WHO) proclaimed the Severe Acute Respiratory Syndrome (SARS) Coronavirus (namely SARS-CoV-2 or COVID-19) as a pandemic in March 2020, there has been much debate regarding the transmission mechanisms of COVID-19.

3. **Line 66: Replace “indoors” with “indoor”**

-Thank you for the comment.

Page 3 (Line 63)

A theoretical model of droplet evaporation is experimentally validated at various temperatures and RH. Based on this model, computational fluid dynamics (CFD) simulations were conducted, coupled with dynamic modelling, to analyse the transport of droplets in an indoor environment.

4. **Line 86: Write PM_2.5_ and PM_10_. Please correct this everywhere in the manuscript.**

- We corrected the term in the manuscript as follows:

Page 4 (Line 85)

The dataset reports concentration levels for air pollutants such as carbon monoxide (CO), nitrogen monoxide (NO), nitrogen dioxide (NO_2_), sulfur dioxide (SO_2_), and particulate matter with aerodynamic diameter ≤ 2.5 μm (PM_2.5_) and ≤ 10 μm (PM_10_) every 6 to 12 hours (depending on the type of pollutant), on a 0.4° x 0.4° latitude and longitude grid.

Page 6 (Line 138)

In addition, we included monthly average precipitation (mm), atmospheric pollutant concentration levels of CO, NO, NO_2_, SO_2_, PM_2.5_ and PM_10_, and three variables provided by the University of Oxford that capture government COVID-19 policies: degree of strictness on closure/containment (0: least strict, 100: most strict), coverage of health resources for contact tracing and vaccine procurement (0: no coverage, 100: highest coverage), and financial support to alleviate economic shock (0: no support, 100: highest support).

5. **Line 114: Replace as “It may be worth explaining about the four….”**

-Thank you for the comment.

Page 5 (Line 115)

It may be worth explaining about the four features of our regression model.

6. **Line 157: Why is** $X_{l,c,t}^{k}$ **bold in equation 1?**

- Thank you for pointing this out. We changed the term to non-bold typeface as follows:

Page 7 (Line 163)

$log (Y_{l,c, t})=\alpha_{c}+\tilde{\alpha}_{c}t+\beta\cdot T_{l,c,t}+\gamma\cdot{RH}_{l,c,t}+\sum_{k} \Theta^{k}X_{l,c,t}^{k}+\varepsilon_{l,c,t}$	(1)

7. **Line 352-359: Authors stated that when d0 is small (e.g. 70 μm), the droplet evaporates in air and becomes an aerosol for all RH cases. This is correct.

   With higher RH the droplet has spread farther and the final height is lower at the fully evaporated state. This cannot be true that with high RH the aerosol has spread farther. The statement looks too general. The droplet will spread farther with low RH where the instant evaporation leads to smaller size aerosols. But, Yes, as compared to d0 of 130 μm, the aerosol of 70 μm can stay longer in air and travel farther with high humidity not as much as of low humidity. There should be clear representation here in order to avoid any misinformation.

   The higher the humidity, the farther the aerosols are dispersed from the source, resulting in a lower local aerosol concentration.
   This should also be corrected here.

   In addition, since the evaporation rate decreases with higher RH, the influence of the gravitational body force increases accordingly, and the droplets fall further to the ground.
   This is true.

   That is, the final aerosol height is lower and further away from the human respiratory tract with higher humidity, resulting in a safer condition.
   But the aerosol will still remain in air and still be infectious. This should not be a way to represent how higher humidity can result in safer condition. However, focus should be on because of higher chances of falling of such aerosols or droplets under high humidity conditions compared to low humidity conditions, one can say higher humidity as a safe condition. Even the 70 μm aerosols at higher humidity can not stay for longer durations as compared to a lower humidity value.**

- Considering the reviewer’s comment, we added additional explanation regarding the differentiation between aerosol and droplet, to prevent further misunderstanding. We defined an aerosol as a droplet that has nearly evaporated. Thus, according to our calculations which utilizes the validated evaporation model, with high RH a small droplet travels farther before fully evaporating into an aerosol. After the droplet has evaporated into an aerosol, it can continue to travel far, but it is at a lower height and farther away from the human respiratory tract, compared to the low RH case. Thus, we believe this likely corresponds to a relatively safer condition.

We have modified the text as follows.

Page 16 (Line 367)

When *d*_0_ is small (e.g. 70 μm), the droplet evaporates in air and becomes an aerosol for all RH cases. With higher RH the droplet (before full evaporation) has spread farther and the final height is lower at the fully evaporated state. On the contrary, after full evaporation, the aerosol is likely to spread farther when RH is low because the advection starts at a higher height than a high RH case. According to previous studies, virus infection by aerosols can occur when one is exposed to a certain concentration over a certain period of time [40]. The higher the humidity, the aerosols are dispersed at a lower height compared to the source, resulting in a lower chance of inhalation since it is further away from the human respiratory tract. In addition, since the evaporation rate decreases with higher RH, the influence of the gravitational body force increases accordingly, and the droplets fall further to the ground. That is, although aerosols can cause infection, with higher humidity the final aerosol height is lower, likely resulting in a relatively safer condition.

8. **Line 484: This is true that higher humidity provides safer environment in terms of COVID protection but a limit should be mentioned here. As we know from previous studies that above 60% RH indoors, there are chances of mold growth and it increases with 80% RH. Be very careful while reporting these values.
   Provide a few literature citations for safer RH values for protecting against both COVID and mold growth here.**

- As the reviewer pointed out, indoor mold growth activates at above 70% relative humidity (Johansson et al. 2010 & Gradeci et al. 2017). Since viruses are also known to survive longer at high (>80%) relative humidity, and human discomfort is strong at high RH, realistically a RH below 70% is recommended.

We have modified the text as below.

Page 22 (Line 482)

An increase in relative humidity had a strong effect on curbing the spread of local cough droplets, and also resulted in a reduction of global COVID-19 transmission. However, all else being equal, temperature neither affected the spread of local cough droplets nor accounted for the global COVID-19 transmission in a statistically significant manner. Our finding is consistent with the fact that COVID-19 has been rapidly spreading both during the summer and winter [22]. Even though we found physical and empirical correlations that enhance our understanding of the spread of COVID-19, we would like to note that finer biological details were not fully considered in this study. For instance, viruses survive for a long time in both low (< 40%) and high (> 80%) relative humidity [49, 50]. It should also be noted that mold growth is activated at above 70% relative humidity and increases with humidity [51, 52].

In summary, we conclude that higher humidity provides a safer environment in terms of virus transmission due to physical respiratory droplet transport. However, safe RH levels should be limited to below 70%, while human discomfort at high humidity levels also needs to be considered when operating indoor HVAC systems, especially for institutions such as hospitals or schools. For future work, natural and hybrid ventilation should be considered since it can influence droplet trajectories.

**Reference**

**[Reviewer 3]**

1. Lin K, Marr LC. Humidity-Dependent Decay of Viruses, but Not Bacteria, in Aerosols and Droplets Follows Disinfection Kinetics. Environ Sci Technol. 2020;54: 1024–1032.

2. Morris DH, Yinda KC, Gamble A, Rossine FW, Huang Q, Bushmaker T, et al. Mechanistic theory predicts the effects of temperature and humidity on inactivation of SARS-CoV-2 and other enveloped viruses. eLife. 2021;10: e65902.

3. Johansson S, Wadso L, Sandin K. Estimation of mould growth levels on rendered facades based on surface relative humidity and surface temperature measurements. Building and Environment. 2010; 45: 1153-1160.

4. Gradeci K, Labonnote N, Time B, Kohler J. Mould growth criteria and design avoidance approaches in wood-based materials – A systematic review. Construction and building material. 2017; 150: 77-88.
