# Supplementary material for: The effects of indoor temperature and humidity on local transmission of COVID-19 and how it relates to global trends
Source: PLoS One. 2022 Aug 10;17(8):e0271760. doi: 10.1371/journal.pone.0271760 (PMC9365153; doi:10.1371/journal.pone.0271760)
Supplement: Attachment — Submitted filename: Response to reviewer #2.docx [file pone.0271760.s005.docx]

**Response to reviewer #2**

We thank the reviewer for the positive feedback. Comments are addressed below.

**1(a). In response to comment #6 in the previous round of review, the authors defined a new characteristic timescale, t_0. The authors mentioned that Figure 2 was modified to reflect this change in the variables defining t_0, but did not elaborate in their reply on how this change in variable affected the final results plotted in Figure 2.**

-Thank you for pointing this out. As reviewer request, previous revision we changed *t_0_* = *D_0_^2^c_p_ρ_l_/k* to *t_0_* = (*D_0_^2^/ Ɗ_g_*)*(ρ_l_/ρ_g_)*, derived from the integral of equation (2), which represents the physical meaning of *t_0_* better. Since the parameters are constant in given environment except *D_0,_* the ratio between two timescales is constant. To elaborate how this change affected Figure 2, we need to compare *D^*2^* before and after the *t_0_* change. As Figure shown below, the value of *t^*^* has increase overall.

**1(b). From analyzing the dimensions of t_0 through the equation provided, the dimensions of t_0 are similar to the dimensions of the diffusion coefficient, not time:**

**((D_0^2)/D_g )(ρ_l/ρ_g ) = t_0
[m^2/s]^2/[m^2/s] ×[kg/m^3 ]/[kg/m^3 ] = [m^2/s]**

**This raises some questions about what the authors claim as a nondimensionalized parameter, t*, because time, t, has units of seconds, s, and t* is defined as t/t0. According to the equations provided, the dimensions for t* will be s2/m2. The authors should consider redefining their characteristic time or should modify their main text to reflect their definition of t*.**

-Thank you for the comment. We checked what the reviewer asked and noticed there was misleading about nomenclature. We used *D_0_* as the droplet diameter, not the diffusion coefficient. Therefore, we found *t_0_* dimension is time [s]. However, we admit that there could be confusion in the nomenclature as both the droplet diameter and diffusion coefficient are written as *D*. To prevent this problem, the diffusion coefficient *D_g_* is modified as *C_Diff_*. This is reflected in the text for clarification.

**((D_0^2)/D_g )(ρ_l/ρ_g ) = t_0
[m]^2/[m^2/s] ×[kg/m^3 ]/[kg/m^3 ] = [s]**

Page 10 (Line 228)

$-\frac{dm_{d}}{dt}=\frac{C_{Diff}}{d}A\rho_{g}Sh\ln\left( 1+B_{m} \right)$	(2)

where $m_{d}$ is droplet mass, *C_Diff_* is the air diffusion coefficient, $d$ is droplet diameter, $A$ is droplet surface area, $\rho_{g}$ is gas density, and $B_{m}=(Y_{s}-Y_{g})/(1-Y_{s})$is the Spalding mass transfer number, where $Y_{s}$ is vapor mass fraction at the droplet surface, and $Y_{g}$ is vapor mass fraction in the ambient gas.

Page 13 (Line 284)

The non-dimensional size is defined as *D*^*^ = *D/D_0_* and non-dimensional time is *t^*^* = *t/t_0_,* where *t_0_* = (*D_0_^2^/C_Diff_*)*(ρ_l_/ρ_g_)*, which is derived from the integral of equation (2). *C_Diff_* is the air diffusion coefficient, *ρ_l_* is liquid (i.e., water) density, and *ρ_g_* is gas density.
