# Supplementary material for: The effects of indoor temperature and humidity on local transmission of COVID-19 and how it relates to global trends
Source: PLoS One. 2022 Aug 10;17(8):e0271760. doi: 10.1371/journal.pone.0271760 (PMC9365153; doi:10.1371/journal.pone.0271760)
Supplement: Attachment — Submitted filename: Park et al. reviewer comments.docx [file pone.0271760.s003.docx]

Park et al., demonstrates the effects of indoor temperature and humidity on local
transmission of COVID-19 and how it relates to global trends. They have thoroughly examined the effect of temperature and relative humidity on respiratory droplet dynamics, and correlated this with COVID-19 transmission. They have validated the droplet evaporation model with experiments performed in an environmental chamber representing indoor conditions. Droplet trajectories were simulated, and they have analyzed how the droplet dynamics vary with temperature and humidity. They have considered two conditions. First, they used indoor conditions since most COVID-transmission is considered to occur indoors. This is the most important part of this study as the authors considered indoor transmission as primary source of COVID related infections. In order to make the connection between indoor physical droplet behavior and global infection trends where outdoor environmental conditions are reported, it is necessary to correlate the indoor and outdoor conditions. In my opinion, this is a very good study which is experimentally and model wise sound. I recommend this study to be published so that governmental bodies and health communities will be benefitted.

However, I have a few comments which needs to be addressed before the final publication.

Line 25: SARS-Cov-2 should be replaced as SARS-CoV-2.

Line 37: Again, please maintain the symmetry while writing SARS-CoV-2.

Line 66: Replace “indoors” with “indoor”.

Line 86: Write PM_2.5_ and PM_10_. Please correct this everywhere in the manuscript.

Line 114: Replace as “It may be worth explaining about the four….”.

Line 157: Why is 𝑿𝒍,𝒄,t bold in equation 1?

Line 352-359: Authors stated that when d0 is small (e.g. 70 μm), the droplet evaporates in air and becomes an aerosol for all RH cases. **This is correct.**

With higher RH the droplet has spread farther and the final height is lower at the fully evaporated state. **This cannot be true that with high RH the aerosol has spread farther. The statement looks too general. The droplet will spread farther with low RH where the instant evaporation leads to smaller size aerosols. But, Yes, as compared to d0 of 130 μm, the aerosol of 70 μm can stay longer in air and travel farther with high humidity not as much as of low humidity. There should be clear representation here in order to avoid any misinformation.**

The higher the humidity, the farther the aerosols are dispersed from the source, resulting in a lower local aerosol concentration.

**This should also be corrected here.**

In addition, since the evaporation rate decreases with higher RH, the influence of the gravitational body force increases accordingly, and the droplets fall further to the ground.

**This is true.**

That is, the final aerosol height is lower and further away from the human respiratory tract with higher humidity, resulting in a safer condition.

**But the aerosol will still remain in air and still be infectious. This should not be a way to represent how higher humidity can result in safer condition. However, focus should be on because of higher chances of falling of such aerosols or droplets under high humidity conditions compared to low humidity conditions, one can say higher humidity as a safe condition. Even the 70 μm aerosols at higher humidity can not stay for longer durations as compared to a lower humidity value.**

Line 484: This is true that higher humidity provides safer environment in terms of COVID protection but a limit should be mentioned here. As we know from previous studies that above 60% RH indoors, there are chances of mold growth and it increases with 80% RH. Be very careful while reporting these values.

Provide a few literature citations for safer RH values for protecting against both COVID and mold growth here.
